# Very High Molecular Weight Hyaluronic Acid as an Enhanced Vehicle in Therapeutic Eye Drops: Application in a Novel Latanoprost Formulation for Glaucoma

**DOI:** 10.3390/bioengineering12090907

**Published:** 2025-08-24

**Authors:** Jesús Pujol-Martí, Wolfgang G. K. Müller-Lierheim

**Affiliations:** i.com Medical GmbH, 81241 Munich, Germany

**Keywords:** hylan A, hyaluronic acid, very high molecular weight, eye drops, ocular drug delivery, ocular surface health, mucoadhesion, viscoelasticity, latanoprost

## Abstract

The efficacy of topical drug delivery via eye drops is often achieved at the expense of tolerability, and consequently, efforts are being made to design strategies that minimize the adverse effects associated with the passage of active pharmaceutical ingredients (APIs) across the ocular surface. Many of these approaches are too complex, costly and challenging to implement on an industrial scale, yet there is increasing evidence that hylan A, a very high molecular weight hyaluronic acid (≥3.0 MDa), may be a promising vehicle for topical drug delivery of ocular therapies. In this review, we explore how the mucoadhesive and viscoelastic properties of eye drop formulations based on hylan A help extend the residence time of APIs at the ocular surface, while maintaining patient comfort. Moreover, we examine how hylan A facilitates the dissolution and stabilization of APIs, as well as their transport across the ocular epithelial barrier, without the need to use toxic penetration enhancers, thereby preserving ocular surface health. Finally, we present evidence indicating that the intrinsic biological properties of hylan A, including its anti-inflammatory effects, help mitigate side effects commonly associated with certain APIs. To illustrate these advantages, we examine the pioneering use of a hylan A-based aqueous eye drop formulation as a vehicle to deliver latanoprost, a prostaglandin analogue widely used in the treatment of glaucoma. This case study demonstrates the potential of hylan A-based eye drops to offer safer and more effective topical drug delivery, especially for long-term ocular therapies where tolerability and biocompatibility are critical.

## 1. Introduction

The anterior segment of the eye comprises the conjunctiva, cornea, and anterior chamber, and it is the primary site of several common ocular disorders. The accessibility of this anterior segment makes it well suited for topical drug delivery, most commonly in the form of eye drops that offer therapeutic opportunities for a range of eye disorders. These approaches offer many advantages, including ease of use, non-invasiveness and lower cost compared to other technologies, establishing eye drops containing active pharmaceutical ingredients (APIs) as first-line therapies for many anterior segment disorders.

However, topical drug delivery via eye drops also faces some limitations, due to the anatomy of the eye and tear dynamics. One challenge is the rapid turnover of the tear film, which causes a large proportion of the eye drop to quickly drain into the nasolacrimal duct, reducing the residence time of the API. The API that resists this initial clearance must still then penetrate the most superficial epithelial barrier and consequently, only a small fraction of the dose applied will ultimately reach deeper structures like the anterior chamber [1,2,3]. A traditional approach to improving topical drug delivery via eye drops has been to include penetration enhancers in the formulations. These facilitate API penetration in different ways: by enhancing penetration of the glycocalyx, corneal and conjunctival surface epithelium; by loosening tight junctions to compromise epithelial integrity; and by altering cell membrane properties. For example, metal chelators like ethylene diamine tetraacetic acid (EDTA) may be used as penetration enhancers as they disrupt the tight junctions between epithelial cells by sequestering calcium ions [4,5].

Eye drop formulations often contain additives to enhance the solubility and stability of the API (e.g., surfactants to dissolve lipophilic APIs), to prolong its retention at the ocular surface (e.g., polymers to enhance viscosity), or to maintain sterility during use and storage (i.e., preservatives). Some additives have adverse effects at the ocular surface, producing irritation, dryness and allergic reactions [6,7]. This is particularly concerning when treating chronic conditions where long-term daily administration is required, and these adverse effects may reduce patient adherence and persistence [8]. By contrast, adverse effects that are associated with the intrinsic molecular properties of the APIs may be less easy to palliate, although they may be alleviated or exacerbated by additives in the formulation [9].

Intense efforts are being made to enhance eye drop formulations for topical drug delivery [10], testing the incorporation of various additives to enhance parameters like API solubility, retention time, and penetration into the eye. However, identifying optimal combinations is still largely considered a challenge, as improvements in one aspect are often either insufficient or may come at the expense of another. For example, preservative-free eye drops produce fewer ocular surface symptoms but they do not appear to be associated with consistently improved patient adherence, for example, in the treatment of glaucoma [11]. This may be due to adverse effects associated with other additives or to the intrinsic properties of the APIs themselves. Hence, there is still a need to develop formulations that enhance API bioavailability while avoiding side effects, and that ideally provide additional benefits to the ocular surface.

Here, we focus on a novel vehicle for topical drug delivery via eye drops: a buffered saline solution containing 0.15% of a linear, very high molecular weight (MW) hyaluronic acid (HA; hylan A, ≥3.0 MDa) [12,13]. This aqueous hylan A formulation offers to address several key challenges, including enhancing ocular surface retention, API solubilisation and stabilization, and API transport, while maintaining a healthy ocular surface. To illustrate these benefits, we will examine the pioneering use of this aqueous hylan A formulation as a vehicle for latanoprost, a prostaglandin analogue and a frontline approach to treat glaucoma.

## 2. An Aqueous Hylan A Formulation with Optimal Viscoelastic Properties Enhances Ocular Surface Retention of APIs

Achieving increased ocular surface retention by adjusting the formulation of eye drops is desirable, as it means the API will stay at the ocular surface longer, thereby enhancing its therapeutic efficacy and drug delivery to the eye. One approach often used to extend ocular surface retention is to increase the viscosity of the eye drop, which hinders drainage from the ocular surface. This can be achieved with synthetic polymers like polyvinyl alcohols (PVAs), natural polymers like HA, or with derivatives of natural polymers such as hypromellose (HPMC) [14]. However, excessively high viscosity can cause problems, like increased reflex tearing, which can accelerate API clearance, exacerbate discomfort and cause temporary visual blurring [14,15]. To avoid these issues, eye drops can be formulated with a polymeric solution that exhibits viscoelastic properties, closely mimicking the flow of healthy human tears. Ideally, such formulations maintain the high viscosity of natural tears when the eye is open, promoting prolonged residence times, while respecting the low viscosity of natural tears during blinking, thereby limiting the friction between the eyelid and the epithelial surface to ensure comfort and avoid altered vision [12,16,17].

**Figure 1:**
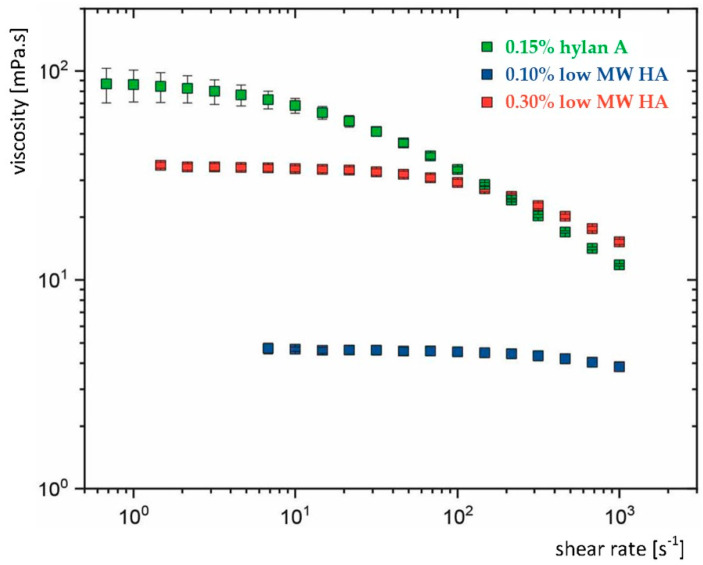
Flow characteristics of three commercial eye drops containing hyaluronic acid (HA) with varying molecular weights (MW) and concentrations. Hylan A: very high MW HA (≥3.0 MDa). Low MW HA eye drops contain HA with average MW ≈ 1.0 MDa. All three eye drops are isotonic, preservative free, and do not contain viscosity-enhancing polymers other than HA. The rheology (dependence of viscosity from shear rate) varies between the three eye drops. Marked viscoelasticity was measured in the eye drops containing hylan A, while viscoelasticity was rare or absent in the eye drops with low MW HA. Adapted from [12].

The flow of dissolved polymers is determined by their ability to entangle [18]. HA is a linear, naturally occurring polysaccharide and an essential component of the vertebrate extracellular matrix [19]. HA can be produced through fermentation, adopting a wide range of MWs (i.e., chain lengths) up to several million Daltons (MDa) [20]. Longer HA chains in solution become more easily entangled, which enhances their viscoelasticity. Accordingly, an aqueous solution with 0.15% hylan A (≥3.0 MDa) exhibits flow behaviour mimicking that of natural tears, whereas an aqueous solution containing lower MW HA does not produce this effect (Figure 1) [12,21]. This is primarily attributed to the very high MW of hylan A without the need for additional viscosity-enhancing agents. It has served as the basis for the development and decades-long commercialisation of hylan A-based eye drops as ocular lubricants (Comfort Shield, i.com medical, Munich, Germany), which have been demonstrated to be a highly biocompatible tear substitute [22,23]. A comprehensive examination of this topic has been presented in a previous article [12].

Mucoadhesion is an even more critical factor than viscosity in determining the retention of APIs at the ocular surface. This feature refers to the interaction with mucus, which is mainly composed of water (>95%) and mucins, a family of large, highly glycosylated hydrophilic proteins [14,24]. Mucins are secreted into the mucoaqueous layer of the tear film at the ocular surface, and they also exist as cell membrane-bound glycoproteins within the glycocalyx formed by the superficial corneal and conjunctival epithelial cells [25]. The flexibility of polymer chains drives their entanglement with mucin chains and the formation of hydrogen bonds [24,26]. In an aqueous solution, HA is a very flexible polyanion able to entangle intimately with and adhere to the mucin molecules at the ocular surface [27]. The mucoadhesive properties of aqueous HA are influenced strongly by its MW; with high MW, HA binds readily to membrane-bound mucins, enhancing the cellular barrier against pathogens and prolonging local drug retention [28,29], but does not with low MW. Indeed, the mucoadhesive performance of linear HA increases in direct proportion with its MW, with crosslinked HA, and other polymers commonly used in eye drops exhibiting lower mucoadhesion [30]. Accordingly, an aqueous hylan A formulation would be expected to exhibit superior mucoadhesiveness at the ocular surface, given its very high MW. Together with its viscoelastic properties comparable to natural tears, an aqueous 0.15% hylan A formulation is therefore likely to be a suitable vehicle for ocular drug delivery, producing adequate ocular surface retention and patient comfort.

## 3. An Aqueous Hylan A Formulation Improves API Solubilisation, Stability and Ocular Transport

A major challenge when using eye drops for topical drug delivery is the poor aqueous solubility and stability of many APIs, which restricts their bioavailability. While advanced solutions have been developed to address this issue [3,15], they are often complex, costly, and may face lengthy evaluation and regulatory approval processes before reaching the market and patients [15,31]. A simpler alternative, supported by extensive biocompatibility and clinical data, is the use of linear, natural HA in an aqueous solution. This approach differs from the use of HA/drug chemical conjugates or HA modified nanoparticles or micelles, although all these strategies exploit similar properties of HA [20,32,33,34,35].

At a physiological pH in an aqueous solution, HA is a negatively charged polyanion and it forms salts, generally referred to as hyaluronan or hyaluronate (e.g., sodium hyaluronate), which are very hydrophilic and, consequently, surrounded by water molecules. More precisely, water molecules link the HA hydrophilic functional groups (e.g., carboxyl, COOH) to hydrogen bonds that stabilize the secondary structure of the biopolymer, i.e., a two-fold helix. In this extended conformation, HA chains also form hydrophobic domains within their secondary structure that can interact non-covalently with other hydrophobic molecules in an aqueous environment [36,37]. This feature can improve the solubility of APIs in aqueous solutions, enhancing their chemical stability by decreasing water accessibility and inhibiting hydrolysis, or by reducing the access of enzymes to the API. When the secondary structure of HA molecules provokes entanglement, an extended three-dimensional network can form (i.e., a tertiary structure), in which the strong intermolecular interactions between HA chains reduce the availability of these hydrophobic domains and the ability of HA to interact with hydrophobic molecules [36]. Both the concentration and MW of HA influence the transition from secondary to tertiary structures in aqueous solutions [20]. Indeed, based on the properties of HA studied previously [36,37], it is plausible that a 0.15% hylan A aqueous solution contains enough HA molecules in conformations that can interact with hydrophobic molecules to improve the solubility and stability of the latter.

**Figure 2:**
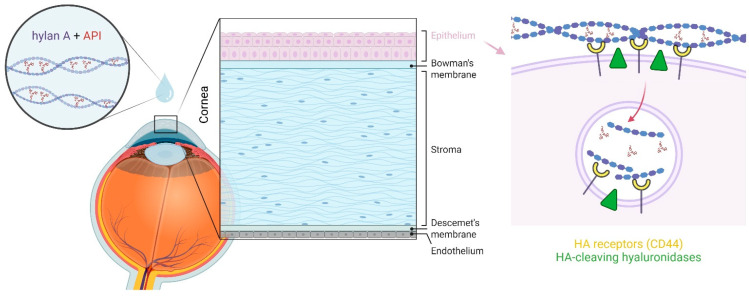
Proposed mechanism by which hylan A acts as a vehicle to transport APIs across the ocular epithelial barrier. Hylan A strongly binds to CD44 receptors on corneal epithelial cells, is cleaved into intermediate-sized fragments by membrane-bound hyaluronidases, and these fragments are internalized and trafficked to intracellular compartments for further degradation. This pathway may enable the delivery of interacting APIs across one of the eye’s primary physiological barriers without compromising cellular or tissue architecture and function. Figure created with BioRender.com.

There is some evidence as to how hylan A might facilitate the transport of other molecules into the eye. In vertebrates, several cell surface receptors for HA exist, the best studied being cluster-determined 44 (CD44) [38,39]. The binding of HA molecules to CD44 involves its engagement with multiple CD44 receptor sites [40], such that the avidity of HA increases with its MW, and high MW HA binds much more strongly to this receptor than low MW forms [41]. Among the known functions of the CD44 receptor is its role in facilitating HA internalization and its subsequent degradation [42]. When high MW HA binds to CD44, it can be cleaved into intermediate-sized fragments by cell surface enzymes (membrane-bound hyaluronidases), which can then be internalized by and directed to intracellular compartments, where they are further degraded [39,43]. CD44 receptors have been identified in both corneal and conjunctival epithelial cells [44,45,46], as this pathway is a potential route for API delivery to deeper ocular regions without compromising cell membrane integrity when these molecules interact with HA (Figure 2). Receptors involved in alternative uptake pathways include the HA receptor for endocytosis (HARE) [47,48], which is also found on corneal epithelial cells [49] and might facilitate the delivery of molecules that interact with HA [12].

## 4. The Benefits of Using an Aqueous Hylan A Formulation for Ocular Surface Health

When the inherent properties of the API in a therapeutic eye drop produce adverse effects, it is important to include substances in these formulations that counteract such effects and that favour ocular surface health. Beyond the benefits outlined above, hylan A has consistently been demonstrated to promote better ocular surface health than lower MW HA, primarily due to its unique very high MW [12].

The MW of HA affects its biological activity in both healthy and diseased states [50,51]. In homeostatic conditions, HA predominantly exists as a high MW polymer with biophysical properties consistent with its activity as a lubricant, space-filler and shock-absorber in joints and connective tissues. Moreover, it has anti-inflammatory, anti-proliferative and anti-angiogenic effects, and multiple studies have highlighted its role as a tissue protectant and promoter of homeostasis after injury and inflammation. By contrast, in pathological circumstances HA fragmentation is enhanced and produces more low MW HA that is linked to inflammation [43,50,51,52]. Furthermore, unlike low MW HA, high MW HA reduces peripheral nociceptor activity [53,54] as well as inflammatory and neuropathic pain [55,56], including pain induced by chemotherapy [57] or surgery [58] in preclinical models. Elsewhere, tear film stability was improved when eye drops with 0.15% aqueous hylan A (Comfort Shield, i.com medical, Munich, Germany) were evaluated in a preclinical model of environmental dry eye stress (Table 1), reducing ocular surface damage and inflammation when compared to the use of low MW HA or secretagogues [59].

Clinical data suggest that the physiological activity of very high MW 0.15% hylan A eye drops is effective to treat severe ocular disease and that they may even be superior to autologous serum eye drops (Table 1) [60]. These benefits were supported by the HYLAN M clinical study, where switching from optimized artificial tear treatments to 0.15% hylan A eye drops significantly improved symptoms in severe dry eye patients (within four weeks), including visual stability, discomfort and pain (Table 1) [23,62,64]. Moreover, 0.15% hylan A eye drops have shown to increase the length of corneal nerves [61], which are typically compromised in this population [65,66,67]. The benefits of hylan A for corneal nerves were further demonstrated in patients who underwent corneal surgery: procedures known to cause unavoidable corneal nerve damage. In this context, daily 0.15% hylan A eye drop application after surgery accelerated the recovery of corneal nerve structure and sensitivity relative to eye drops containing low MW HA, while also improving ocular surface symptoms [63]. In addition, hylan A treatment helped prevent the increase in inflammation-related immune cells observed three months after surgery when low MW HA was used (Table 1) [63].

## 5. An Aqueous Hylan A Formulation as a New Vehicle for Latanoprost to Manage Elevated Intraocular Pressure

Alterations to the anterior segment may influence the development of diseases that affect structures in the posterior eye segment, such as the retina and optic nerve. One prominent example is glaucoma, one of the most common worldwide causes of irreversible blindness. Glaucoma often develops when aqueous humour drainage from the anterior chamber is impaired or unbalanced, leading to an elevation in intraocular pressure (IOP) that can provoke structural changes in the posterior segment of the eye and optic nerve injury. Currently, IOP is the only risk factor that can be modulated to prevent glaucoma progression [11,68]. Thus, reducing IOP via eye drops containing APIs is the primary strategy to treat glaucoma [11].

Prostaglandin analogues increase aqueous humour outflow via the unconventional pathway, such that latanoprost has become a frequently used treatment [11] and one of the most effective APIs used in eye drops to lower IOP and slow glaucoma progression [69]. Prostaglandin analogues produce well documented adverse effects at the ocular surface [70] and at therapeutic concentrations; topical latanoprost can cause ocular surface damage in a mouse model that resembles dry eye disease, primarily through inflammatory mechanisms [71]. Moreover, many commercial IOP-lowering eye drops contain the quaternary ammonium cationic detergent benzalkonium chloride (BAK) as a preservative and penetration enhancer [6], although long-term BAK use has negative effects at the ocular surface [72] and an emergence of preservative-free IOP-lowering eye drops has occurred in recent years [6,73,74,75]. These formulations may include other penetration enhancers like EDTA, which, while less problematic, can potentially cause long-term ocular surface damage [5,76,77]. Other additives like polyethylene glycol (PEG) and propylene glycol (PG) act as lubricants, surfactants, and co-solubilisers, yet they may also contribute to dry eye disease [78].

The prevalence of ocular surface disease is very high in individuals with glaucoma, particularly in those with uncontrolled glaucoma and those who use multiple topical medications [79,80,81]. A large-scale study found a higher IOP in glaucoma patients with severe ocular surface disease than in those with no or mild disease [81]. Moreover, there is evidence that chronic ocular surface inflammation increases outflow resistance, further favouring IOP elevation [82,83,84]. Therefore, adopting a management strategy for ocular surface problems, particularly inflammation, is key to improving the effectiveness of IOP control. This can be achieved by reducing the toxicity of glaucoma medications and incorporating supportive therapies (e.g., ocular surface lubrication and anti-inflammatory treatment) [85,86,87].

To address these challenges, a novel latanoprost ophthalmic formulation has been developed using an aqueous hylan A solution as the vehicle, leveraging the advantages outlined in the previous sections. The formulation is a preservative-free 0.15% hylan A solution prepared in isotonic phosphate-buffered saline at pH 7.4: a stable vehicle, to which 20 μg/mL of latanoprost is added [88]. Notably, this concentration exceeds the normal solubility of latanoprost in water by approximately 8 μg/mL (Table 2), probably facilitated by an interaction between latanoprost and hylan A. In one subject with ocular hypertension, this new formulation lowered the IOP more than a commercial latanoprost eye drop (Table 2), despite the higher concentration in the latter (50 μg/mL latanoprost) [88]. This effect further suggests that hylan A may facilitate the transport of latanoprost into the eye [12,13], as seen in a rat model where the therapeutic concentration of latanoprost in the animals’ aqueous humour was similar after the administration of a hylan A-based eye drop containing 14 μg/mL latanoprost to that achieved with a commercial formulation containing 50 μg/mL latanoprost (Table 2). Indeed, this commercial formulation not only contained around 3.5 times more latanoprost, but it also included the penetration enhancer EDTA [89]. The same formulations were also tested in a mouse model, in which the novel hylan A-based formulation induced less inflammation, caused fewer ocular surface alterations, and retained better corneal epithelial barrier integrity than the commercial formulation, while controlling IOP better. Significantly, all the ocular surface parameters analyzed in animals treated with the hylan A-based latanoprost formulation were indistinguishable from those of the untreated wild-type controls (Table 2) [90].

Taken together, these findings support the potential of this novel latanoprost formulation, which is free of toxic additives, that uses a lower amount of the API, and that takes advantage of the lubricating and anti-inflammatory properties of hylan A. By promoting ocular surface health, this formulation may enhance the reduction of IOP by chronic treatments, as preclinical results indicate. Further clinical studies are being prepared to confirm these potential benefits.

## 6. Hylan A-Based Eye Drops as Next Generation Vehicles for Therapeutic API Delivery

A variety of new delivery systems for topical drugs are being developed that offer promise to advance ocular therapy, such as hydrogels, nanoparticle carriers [91], contact lenses and ocular inserts [92]. However, they pose significant challenges related to implantation, removal, long-term biocompatibility, and patient acceptance. Many of these new technologies are also costly and difficult to scale up for industrial production. In the case of glaucoma, which affects around 100 million people worldwide across a wide range of socioeconomic and healthcare settings [70], it is essential to establish a range of treatment options. Thus, eye drops continue to represent a practical, accessible and widely accepted option.

There are many ongoing efforts to improve eye drop formulations for topical drug delivery, with different additives being tested to enhance critical parameters like mucoadhesiveness, viscoelasticity and API solubility, stability and ocular transport, as well as to favour ocular surface health. While optimizing one parameter in many new formulations compromises another, the hylan A-based eye drops presented here seem to represent a promising solution to simultaneously address all these critical aspects. Indeed, the evidence supports the efficacy of hylan A-based eye drops to treat various ocular surface conditions (Table 1), and also as a useful vehicle for latanoprost (Table 2). Although the benefits observed can mostly be attributed to the unique properties of hylan A, other physicochemical properties of the formulation also contribute positively, such as pH, osmolarity and the buffer employed [9,89].

We envisage that hylan A-based eye drops will represent a platform that could support a wide range of ocular therapies. A further promising application is the topical delivery of cyclosporine A (CsA), an immunosuppressive drug commonly used to manage the inflammatory component of chronic dry eye disease [93,94]. This is a condition where effectively overcoming ocular drug delivery barriers while avoiding adverse effects remains a challenge, mainly due to the large MW and hydrophobic nature of CsA [95,96]. Hylan A-based eye drops could address these limitations, as already seen with latanoprost [88,89,90]. Beyond its use as a vehicle, hylan A itself produces anti-inflammatory effects [13,59] that may act synergistically with CsA to more effectively treat chronic dry eye disease. Moreover, given the involvement of neurosensory abnormalities in dry eye disease [94], the neurotrophic effects of hylan A [61,63] may offer further therapeutic benefits. Interestingly, recent findings in vitro showed that CsA can alter HA metabolism in orbital fibroblasts [97]. If similar effects occur in vivo, this could have negative effects on ocular surface health. In such a case, co-formulation of CsA with hylan A may help maintain physiological HA homeostasis and mitigate potential adverse effects.

In addition to enhancing the ocular delivery of hydrophobic APIs, hylan A-based eye drop technology also shows promise in improving the efficacy and tolerability of treatments using APIs that are highly soluble in aqueous solutions. In line with this, the hylan A-based aqueous formulation described here is currently being investigated as a vehicle for ketotifen, a widely used antihistamine used in allergic conjunctivitis. Recent data presented at the tear film and ocular surface society (TFOS) meeting in 2024 showed that, in a mouse model of allergic conjunctivitis, the novel hylan A-based ketotifen aqueous formulation improves ocular surface status and maintains anti-allergic efficacy compared with commercial ketotifen eye drops [98].

Certain limitations on using hylan A as a vehicle in therapeutic eye drops should also be considered. Thus far, data demonstrating solubility and stability enhancement for hydrophobic APIs are limited to latanoprost (Table 2). For larger molecules, such as CsA, the hylan A-based aqueous formulation might not suffice and an additional excipient may be needed. Furthermore, there are currently no clinical data from human trials, and the evidence is limited to preclinical investigations (Table 2). Clinical trials comparing hylan A-based formulations with current standard therapies are necessary to validate their efficacy, safety and tolerability in real-world settings. Finally, while preclinical results indicate enhanced delivery of APIs to the anterior segment of the eye (Table 2), it remains unclear whether the presented formulation can transport APIs to more posterior regions, which may limit its utility for certain medical indications.

In summary, the use of hylan A as a vehicle represents a promising step towards a new generation of eye drops for topical ocular drug delivery, designed to improve API bioavailability, reduce adverse effects, and support long-term ocular surface health in a range of therapeutic indications.

## Figures and Tables

**Table 1:** Summary of the studies demonstrating the benefits of the 0.15% hylan A eye drop formulations in ocular surface health.

Study	Study Type	Model/ Patients	Comparisons	Conclusions
Kojima et al., 2020 [59]	Preclinical	Mouse model of environmental dry eye disease	Low MW HA eye drops, secretagogue eye drops	Improved tear film stability. Reduced ocular surface damage. Less inflammation with 0.15% hylan A eye drops.
Beck et al., 2019 [60]	Clinical	11 patients treated with autologous serum eye drops	Autologous serum eye drops	Eye drops containing 0.15% hylan A effectively treat severe ocular disease. These 0.15% hylan A eye drops may replace eye drops with autologous serum.
van Setten et al., 2020a [23]	Clinical HYLAN M study	84 patients with severe dry eye disease	Optimized artificial tear treatments	Symptoms rapidly improved by switching to 0.15% hylan A eye drops include: visual stability, discomfort and pain.
van Setten et al., 2020b [61]	Clinical. Subgroup analysis of the HYLAN M study	16 patients	Optimized artificial tear treatments	Switching to 0.15% hylan A eye drops promotes corneal nerve growth.
Medic et al., 2024 [62]	Clinical Subgroup analysis of the HYLAN M study	47 patients	HA containing artificial tears (15 commercial brands with HA of diverse MWs)	Fewer 0.15% hylan A eye drops required than those with lower MW HA. Eye drops containing 0.15% hylan A have better clinical effects.
Özkan et al., 2025 [63]	Clinical	63 eyes from 55 patients with keratoconus following corneal crosslinking (CXL)	Low MW HA eye drops	After CXL, 0.15% hylan A eye drops produce: faster corneal nerve regeneration; faster recovery of sensitivity; improved ocular symptoms; and fewer inflammation-related immune cells than low MW HA eye drops.

**Table 2:** Summary of the studies demonstrating the benefits of a novel 0.15% hylan A eye drop formulation as a vehicle for latanoprost.

Study	Study Type	Model/ Patients	Comparators	Conclusions
Müller-Lierheim, 2021 [88]	Formulation solubility and stability	N/A	N/A	A preservative-free 0.15% hylan A solution in isotonic phosphate-buffered saline (pH 7.4) enhances latanoprost solubility by 75%
Dogru et al., 2023 [90]	Preclinical	Standard mouse strain	Commercial eye drops with 50 μg/mL latanoprost	A hylan A-based eye drop with 14 μg/mL latanoprost preserves ocular surface parameters while effectively lowering IOP
Higa et al., 2024 [89]	Preclinical	Standard strain rat	Commercial eye drops with 50 μg/mL latanoprost	A hylan A-based eye drop with 14 μg/mL latanoprost achieves comparable therapeutic latanoprost levels in aqueous humour as a 50 μg/mL commercial formulation
Müller-Lierheim, 2021 [88]	Proof-of-concept	One subject with ocular hypertension	Commercial eye drops with 50 μg/mL latanoprost	A hylan A-based eye drop with 20 μg/mL latanoprost lowers IOP better than a commercial latanoprost eye drop with a higher API concentration

## References

[1] Allyn M.M., Luo R.H., Hellwarth E.B., Swindle-Reilly K.E. (2021). Considerations for Polymers Used in Ocular Drug Delivery. Front. Med..

[2] Hansen M.E., Ibrahim Y., Desai T.A., Koval M. (2024). Nanostructure-Mediated Transport of Therapeutics through Epithelial Barriers. Int. J. Mol. Sci..

[3] Ahmed S., Amin M.M., Sayed S. (2023). Ocular Drug Delivery: A Comprehensive Review. AAPS PharmSciTech.

[4] Moiseev R.V., Morrison P.W.J., Steele F., Khutoryanskiy V.V. (2019). Penetration Enhancers in Ocular Drug Delivery. Pharmaceutics.

[5] Ye J., Wu H., Wu Y., Wang C., Zhang H., Shi X., Yang J. (2012). High molecular weight hyaluronan decreases oxidative DNA damage induced by EDTA in human corneal epithelial cells. Eye.

[6] Kahook M.Y., Rapuano C.J., Messmer E.M., Radcliffe N.M., Galor A., Baudouin C. (2024). Preservatives and ocular surface disease: A review. Ocul. Surf..

[7] Fineide F., Magno M., Dahlo K., Kolko M., Heegaard S., Vehof J., Utheim T.P. (2024). Topical glaucoma medications—Possible implications on the meibomian glands. Acta Ophthalmol..

[8] Baudouin C., Myers J.S., Van Tassel S.H., Goyal N.A., Martinez-de-la-Casa J., Ng A., Evans J.S. (2025). Adherence and Persistence on Prostaglandin Analogues for Glaucoma: A Systematic Review and Meta-Analysis. Am. J. Ophthalmol..

[9] Hedengran A., Kolko M. (2023). The molecular aspect of anti-glaucomatous eye drops—Are we harming our patients?. Mol. Aspects Med..

[10] Wang Y., Wang C. (2022). Novel Eye Drop Delivery Systems: Advance on Formulation Design Strategies Targeting Anterior and Posterior Segments of the Eye. Pharmaceutics.

[11] Jayaram H., Kolko M., Friedman D.S., Gazzard G. (2023). Glaucoma: Now and beyond. Lancet.

[12] Müller-Lierheim W.G.K. (2020). Why Chain Length of Hyaluronan in Eye Drops Matters. Diagnostics.

[13] Bron A.J., Dogru M., Horwath-Winter J., Kojima T., Kovacs I., Muller-Lierheim W.G.K., van Setten G.B., Belmonte C. (2022). Reflections on the Ocular Surface: Summary of the Presentations at the 4th Coronis Foundation Ophthalmic Symposium Debate: “A Multifactorial Approach to Ocular Surface Disorders” (August 31 2021). Front. Biosci..

[14] Grassiri B., Zambito Y., Bernkop-Schnurch A. (2021). Strategies to prolong the residence time of drug delivery systems on ocular surface. Adv. Colloid Interface Sci..

[15] Giri B.R., Jakka D., Sandoval M.A., Kulkarni V.R., Bao Q. (2024). Advancements in Ocular Therapy: A Review of Emerging Drug Delivery Approaches and Pharmaceutical Technologies. Pharmaceutics.

[16] Arshinoff S., Hofmann I., Nae H. (2021). Role of rheology in tears and artificial tears. J. Cataract Refract. Surg..

[17] Tiffany J.M. (1994). Viscoelastic properties of human tears and polymer solutions. Adv. Exp. Med. Biol..

[18] Graessley W.W. (2005). The Entanglement Concept in Polymer Rheology.

[19] Almond A. (2007). Hyaluronan. Cell. Mol. Life Sci..

[20] Fallacara A., Baldini E., Manfredini S., Vertuani S. (2018). Hyaluronic Acid in the Third Millennium. Polymers.

[21] Arshinoff S., Hofmann I., Nae H. (2021). Rheological behavior of commercial artificial tear solutions. J. Cataract Refract. Surg..

[22] Polack F.M., McNiece M. (1982). The treatment of dry eyes with Na hyaluronate (Healon^®^). Cornea.

[23] van Setten G.B., Baudouin C., Horwath-Winter J., Bohringer D., Stachs O., Toker E., Al-Zaaidi S., Benitez-Del-Castillo J.M., Beck R., Al-Sheikh O. (2020). The HYLAN M Study: Efficacy of 0.15% High Molecular Weight Hyaluronan Fluid in the Treatment of Severe Dry Eye Disease in a Multicenter Randomized Trial. J. Clin. Med..

[24] Andrews G.P., Laverty T.P., Jones D.S. (2009). Mucoadhesive polymeric platforms for controlled drug delivery. Eur. J. Pharm. Biopharm..

[25] Baudouin C., Rolando M., Benitez Del Castillo J.M., Messmer E.M., Figueiredo F.C., Irkec M., Van Setten G., Labetoulle M. (2019). Reconsidering the central role of mucins in dry eye and ocular surface diseases. Prog. Retin. Eye Res..

[26] Smart J.D. (2005). The basics and underlying mechanisms of mucoadhesion. Adv. Drug Deliv. Rev..

[27] Zhang X., Wei D., Xu Y., Zhu Q. (2021). Hyaluronic acid in ocular drug delivery. Carbohydr. Polym..

[28] Hansen I.M., Ebbesen M.F., Kaspersen L., Thomsen T., Bienk K., Cai Y., Malle B.M., Howard K.A. (2017). Hyaluronic Acid Molecular Weight-Dependent Modulation of Mucin Nanostructure for Potential Mucosal Therapeutic Applications. Mol. Pharm..

[29] Durrani A.M., Farr S.J., Kellaway I.W. (1995). Influence of molecular weight and formulation pH on the precorneal clearance rate of hyaluronic acid in the rabbit eye. Int. J. Pharm..

[30] Guarise C., Acquasaliente L., Pasut G., Pavan M., Soato M., Garofolin G., Beninatto R., Giacomel E., Sartori E., Galesso D. (2023). The role of high molecular weight hyaluronic acid in mucoadhesion on an ocular surface model. J. Mech. Behav. Biomed. Mater..

[31] Tenpattinam S.S., Bukke S.P.N., Kusuma P.K., Onohuean H., Mothilal M., Krishnamaraju U., Goruntla N., Yadesa T.M. (2025). Self-assembled nanoparticles in ocular delivery: A comprehensive review. Discov. Appl. Sci..

[32] Jiang H., Xu Z. (2023). Hyaluronic acid-based nanoparticles to deliver drugs to the ocular posterior segment. Drug Deliv..

[33] Zhang Y., Sun T., Jiang C. (2018). Biomacromolecules as carriers in drug delivery and tissue engineering. Acta Pharm. Sin. B.

[34] Guter M., Breunig M. (2017). Hyaluronan as a promising excipient for ocular drug delivery. Eur. J. Pharm. Biopharm..

[35] Buckley C., Murphy E.J., Montgomery T.R., Major I. (2022). Hyaluronic Acid: A Review of the Drug Delivery Capabilities of This Naturally Occurring Polysaccharide. Polymers.

[36] Rouse J.J., Whateley T.L., Thomas M., Eccleston G.M. (2007). Controlled drug delivery to the lung: Influence of hyaluronic acid solution conformation on its adsorption to hydrophobic drug particles. Int. J. Pharm..

[37] Ghosh P., Hutadilok N., Adam N., Lentini A. (1994). Interactions of hyaluronan (hyaluronic acid) with phospholipids as determined by gel permeation chromatography, multi-angle laser-light-scattering photometry and 1H-NMR spectroscopy. Int. J. Biol. Macromol..

[38] Aruffo A., Stamenkovic I., Melnick M., Underhill C.B., Seed B. (1990). CD44 is the principal cell surface receptor for hyaluronate. Cell.

[39] Wang X., Liu X., Li C., Li J., Qiu M., Wang Y., Han W. (2025). Effects of molecular weights on the bioactivity of hyaluronic acid: A review. Carbohydr. Res..

[40] Ruppert S.M., Hawn T.R., Arrigoni A., Wight T.N., Bollyky P.L. (2014). Tissue integrity signals communicated by high-molecular weight hyaluronan and the resolution of inflammation. Immunol. Res..

[41] Lee-Sayer S.S., Dong Y., Arif A.A., Olsson M., Brown K.L., Johnson P. (2015). The where, when, how, and why of hyaluronan binding by immune cells. Front. Immunol..

[42] Knudson W., Chow G., Knudson C.B. (2002). CD44-mediated uptake and degradation of hyaluronan. Matrix Biol..

[43] Garantziotis S., Savani R.C. (2019). Hyaluronan biology: A complex balancing act of structure, function, location and context. Matrix Biol..

[44] Lardner E., van Setten G.B. (2020). Detection of TSG-6-like protein in human corneal epithelium. Simultaneous presence with CD44 and hyaluronic acid. J. Fr. Ophtalmol..

[45] Zhu S.N., Nolle B., Duncker G. (1997). Expression of adhesion molecule CD44 on human corneas. Br. J. Ophthalmol..

[46] Lerner L.E., Schwartz D.M., Hwang D.G., Howes E.L., Stern R. (1998). Hyaluronan and CD44 in the human cornea and limbal conjunctiva. Exp. Eye Res..

[47] Zhou B., Weigel J.A., Fauss L., Weigel P.H. (2000). Identification of the hyaluronan receptor for endocytosis (HARE). J. Biol. Chem..

[48] Harris E.N., Baker E. (2020). Role of the Hyaluronan Receptor, Stabilin-2/HARE, in Health and Disease. Int. J. Mol. Sci..

[49] Falkowski M., Schledzewski K., Hansen B., Goerdt S. (2003). Expression of stabilin-2, a novel fasciclin-like hyaluronan receptor protein, in murine sinusoidal endothelia, avascular tissues, and at solid/liquid interfaces. Histochem. Cell Biol..

[50] Bohaumilitzky L., Huber A.K., Stork E.M., Wengert S., Woelfl F., Boehm H. (2017). A Trickster in Disguise: Hyaluronan’s Ambivalent Roles in the Matrix. Front. Oncol..

[51] Cyphert J.M., Trempus C.S., Garantziotis S. (2015). Size Matters: Molecular Weight Specificity of Hyaluronan Effects in Cell Biology. Int. J. Cell Biol..

[52] Monslow J., Govindaraju P., Pure E. (2015). Hyaluronan—A functional and structural sweet spot in the tissue microenvironment. Front. Immunol..

[53] Gomis A., Pawlak M., Balazs E.A., Schmidt R.F., Belmonte C. (2004). Effects of different molecular weight elastoviscous hyaluronan solutions on articular nociceptive afferents. Arthritis Rheum..

[54] Caires R., Luis E., Taberner F.J., Fernandez-Ballester G., Ferrer-Montiel A., Balazs E.A., Gomis A., Belmonte C., de la Pena E. (2015). Hyaluronan modulates TRPV1 channel opening, reducing peripheral nociceptor activity and pain. Nat. Commun..

[55] Bonet I.J.M., Araldi D., Khomula E.V., Bogen O., Green P.G., Levine J.D. (2020). Mechanisms Mediating High-Molecular-Weight Hyaluronan-Induced Antihyperalgesia. J. Neurosci..

[56] Ferrari L.F., Khomula E.V., Araldi D., Levine J.D. (2018). CD44 Signaling Mediates High Molecular Weight Hyaluronan-Induced Antihyperalgesia. J. Neurosci..

[57] Bonet I.J.M., Staurengo-Ferrari L., Araldi D., Green P.G., Levine J.D. (2022). Second messengers mediating high-molecular-weight hyaluronan-induced antihyperalgesia in rats with chemotherapy-induced peripheral neuropathy. Pain.

[58] Zhang C., Huang Q., Ford N.C., Limjunyawong N., Lin Q., Yang F., Cui X., Uniyal A., Liu J., Mahabole M. (2024). Human birth tissue products as a non-opioid medicine to inhibit post-surgical pain. eLife.

[59] Kojima T., Nagata T., Kudo H., Muller-Lierheim W.G.K., van Setten G.B., Dogru M., Tsubota K. (2020). The Effects of High Molecular Weight Hyaluronic Acid Eye Drop Application in Environmental Dry Eye Stress Model Mice. Int. J. Mol. Sci..

[60] Beck R., Stachs O., Koschmieder A., Mueller-Lierheim W.G.K., Peschel S., van Setten G.B. (2019). Hyaluronic Acid as an Alternative to Autologous Human Serum Eye Drops: Initial Clinical Results with High-Molecular-Weight Hyaluronic Acid Eye Drops. Case Rep. Ophthalmol..

[61] van Setten G.B., Stachs O., Dupas B., Turhan S.A., Seitz B., Reitsamer H., Winter K., Horwath-Winter J., Guthoff R.F., Muller-Lierheim W.G.K. (2020). High Molecular Weight Hyaluronan Promotes Corneal Nerve Growth in Severe Dry Eyes. J. Clin. Med..

[62] Medic N., Boldin I., Berisha B., Matijak-Kronschachner B., Aminfar H., Schwantzer G., Muller-Lierheim W.G.K., van Setten G.B., Horwath-Winter J. (2024). Application frequency—Key indicator for the efficiency of severe dry eye disease treatment—Evidence for the importance of molecular weight of hyaluronan in lubricating agents. Acta Ophthalmol..

[63] Özkan G., Turhan S.A., Toker E. (2025). Effect of high and low molecular weight sodium hyaluronic acid eye drops on corneal recovery after crosslinking in keratoconus patients. BMJ Open Ophthalmol..

[64] Alsheikh O., Alzaaidi S., Vargas J.M., Al-Sharif E., Alrajeh M., AlSemari M.A., Alhommadi A., Alsaati A., Aljwaiser N., Alshahwan E. (2021). Effectiveness of 0.15% hylan A eye drops in ameliorating symptoms of severe dry eye patients in Saudi Arabia. Saudi J. Ophthalmol..

[65] Benitez-Del-Castillo J.M., Acosta M.C., Wassfi M.A., Diaz-Valle D., Gegundez J.A., Fernandez C., Garcia-Sanchez J. (2007). Relation between corneal innervation with confocal microscopy and corneal sensitivity with noncontact esthesiometry in patients with dry eye. Invest. Ophthalmol. Vis. Sci..

[66] Shetty R., Dua H.S., Tong L., Kundu G., Khamar P., Gorimanipalli B., D’Souza S. (2023). Role of in vivo confocal microscopy in dry eye disease and eye pain. Indian J. Ophthalmol..

[67] Galor A., Gallar J., Acosta M.C., Meseguer V., Benitez-Del-Castillo J.M., Stachs O., Szentmary N., Versura P., Muller-Lierheim W.G.K., Belmonte C. (2025). CORONIS symposium 2023: Scientific and clinical frontiers in ocular surface innervation. Acta Ophthalmol..

[68] Weinreb R.N., Aung T., Medeiros F.A. (2014). The pathophysiology and treatment of glaucoma: A review. JAMA.

[69] Li T., Lindsley K., Rouse B., Hong H., Shi Q., Friedman D.S., Wormald R., Dickersin K. (2016). Comparative Effectiveness of First-Line Medications for Primary Open-Angle Glaucoma: A Systematic Review and Network Meta-analysis. Ophthalmology.

[70] Kolko M., Gazzard G., Baudouin C., Beier S., Brignole-Baudouin F., Cvenkel B., Fineide F., Hedengran A., Hommer A., Jespersen E. (2023). Impact of glaucoma medications on the ocular surface and how ocular surface disease can influence glaucoma treatment. Ocul. Surf..

[71] Yang Y., Huang C., Lin X., Wu Y., Ouyang W., Tang L., Ye S., Wang Y., Li W., Zhang X. (2018). 0.005% Preservative-Free Latanoprost Induces Dry Eye-Like Ocular Surface Damage via Promotion of Inflammation in Mice. Invest. Ophthalmol. Vis. Sci..

[72] Baudouin C., Labbe A., Liang H., Pauly A., Brignole-Baudouin F. (2010). Preservatives in eyedrops: The good, the bad and the ugly. Prog. Retin. Eye Res..

[73] Konstas A.G., Labbe A., Katsanos A., Meier-Gibbons F., Irkec M., Boboridis K.G., Hollo G., Garcia-Feijoo J., Dutton G.N., Baudouin C. (2021). The treatment of glaucoma using topical preservative-free agents: An evaluation of safety and tolerability. Expert Opin. Drug Saf..

[74] Hollo G., Katsanos A., Boboridis K.G., Irkec M., Konstas A.G.P. (2018). Preservative-Free Prostaglandin Analogs and Prostaglandin/Timolol Fixed Combinations in the Treatment of Glaucoma: Efficacy, Safety and Potential Advantages. Drugs.

[75] Kim J.M., Park S.W., Seong M., Ha S.J., Lee J.W., Rho S., Lee C.E., Kim K.N., Kim T.W., Sung K.R. (2021). Comparison of the Safety and Efficacy between Preserved and Preservative-Free Latanoprost and Preservative-Free Tafluprost. Pharmaceuticals.

[76] Villani E., Sacchi M., Magnani F., Nicodemo A., Williams S.E., Rossi A., Ratiglia R., De Cilla S., Nucci P. (2016). The Ocular Surface in Medically Controlled Glaucoma: An In Vivo Confocal Study. Invest. Ophthalmol. Vis. Sci..

[77] Halder A., Khopade A.J. (2020). Physiochemical Properties and Cytotoxicity of a Benzalkonium Chloride-Free, Micellar Emulsion Ophthalmic Formulation of Latanoprost. Clin. Ophthalmol..

[78] Gomes J.A.P., Azar D.T., Baudouin C., Efron N., Hirayama M., Horwath-Winter J., Kim T., Mehta J.S., Messmer E.M., Pepose J.S. (2017). TFOS DEWS II iatrogenic report. Ocul. Surf..

[79] Fechtner R.D., Godfrey D.G., Budenz D., Stewart J.A., Stewart W.C., Jasek M.C. (2010). Prevalence of ocular surface complaints in patients with glaucoma using topical intraocular pressure-lowering medications. Cornea.

[80] Skalicky S.E., Goldberg I., McCluskey P. (2012). Ocular surface disease and quality of life in patients with glaucoma. Am. J. Ophthalmol..

[81] Baudouin C., Renard J.P., Nordmann J.P., Denis P., Lachkar Y., Sellem E., Rouland J.F., Jeanbat V., Bouee S. (2013). Prevalence and risk factors for ocular surface disease among patients treated over the long term for glaucoma or ocular hypertension. Eur. J. Ophthalmol..

[82] Baudouin C., Denoyer A., Desbenoit N., Hamm G., Grise A. (2012). In vitro and in vivo experimental studies on trabecular meshwork degeneration induced by benzalkonium chloride (an American Ophthalmological Society thesis). Trans. Am. Ophthalmol. Soc..

[83] Batra R., Tailor R., Mohamed S. (2014). Ocular surface disease exacerbated glaucoma: Optimizing the ocular surface improves intraocular pressure control. J. Glaucoma.

[84] Baudouin C., Kolko M., Melik-Parsadaniantz S., Messmer E.M. (2021). Inflammation in Glaucoma: From the back to the front of the eye, and beyond. Prog. Retin. Eye Res..

[85] Dubrulle P., Labbe A., Brasnu E., Liang H., Hamard P., Meziani L., Baudouin C. (2018). Influence of Treating Ocular Surface Disease on Intraocular Pressure in Glaucoma Patients Intolerant to Their Topical Treatments: A Report of 10 Cases. J. Glaucoma.

[86] Messmer E.M., Baudouin C., Benitez-Del-Castillo J.M., Iester M., Anton A., Thygesen J., Topouzis F. (2024). Expert Consensus Recommendations for the Management of Ocular Surface Inflammation in Patients With Glaucoma. J. Glaucoma.

[87] Kemer O.E., Mekala P., Dave B., Kooner K.S. (2024). Managing Ocular Surface Disease in Glaucoma Treatment: A Systematic Review. Bioengineering.

[88] Müller-Lierheim W.G. (2021). Hylan a: A novel transporter for Latanoprost in the treatment of ocular hypertension. Biomed. J. Sci. Tech. Res..

[89] Higa K., Kimoto R., Kojima T., Dogru M., Muller-Lierheim W.G.K., Shimazaki J. (2024). Therapeutic Aqueous Humor Concentrations of Latanoprost Attained in Rats by Administration in a Very-High-Molecular-Weight Hyaluronic Acid Eye Drop. Pharmaceutics.

[90] Dogru M., Kojima T., Higa K., Igarashi A., Kudo H., Muller-Lierheim W.G.K., Tsubota K., Negishi K. (2023). The Effect of High Molecular Weight Hyaluronic Acid and Latanoprost Eyedrops on Tear Functions and Ocular Surface Status in C57/BL6 Mice. J. Clin. Med..

[91] Yang C.J., Nguyen D.D., Lai J.Y. (2023). Poly(l-Histidine)-Mediated On-Demand Therapeutic Delivery of Roughened Ceria Nanocages for Treatment of Chemical Eye Injury. Adv. Sci..

[92] Zeppieri M., Gagliano C., Tognetto D., Musa M., Rossi F.B., Greggio A., Gualandi G., Galan A., Babighian S. (2025). Unraveling the Mechanisms, Clinical Impact, Comparisons, and Safety Profiles of Slow-Release Therapies in Glaucoma. Pharmaceutics.

[93] Patil S., Sawale G., Ghuge S., Sathaye S. (2025). Quintessence of currently approved and upcoming treatments for dry eye disease. Graefes Arch. Clin. Exp. Ophthalmol..

[94] Craig J.P., Nichols K.K., Akpek E.K., Caffery B., Dua H.S., Joo C.K., Liu Z., Nelson J.D., Nichols J.J., Tsubota K. (2017). TFOS DEWS II Definition and Classification Report. Ocul. Surf..

[95] Nagai N., Otake H. (2022). Novel drug delivery systems for the management of dry eye. Adv. Drug Deliv. Rev..

[96] Periman L.M., Mah F.S., Karpecki P.M. (2020). A Review of the Mechanism of Action of Cyclosporine A: The Role of Cyclosporine A in Dry Eye Disease and Recent Formulation Developments. Clin. Ophthalmol..

[97] Galgoczi E., Molnar Z., Katko M., Ujhelyi B., Steiber Z., Nagy E.V. (2024). Cyclosporin A inhibits PDGF-BB induced hyaluronan synthesis in orbital fibroblasts. Chem. Biol. Interact..

[98] Dogru M., Nagata T., Kojima T., Higa K., Okada N., Müller-Lierheim W., Fukagawa K., Fujishima H., Negishi K. The effect of a novel high molecular weight hyaluronic acid and ketotifen eye drop on the ocular surface status in an allergic conjunctivitis mouse model. Proceedings of the TFOS 2024 Conference.

